# Renal Effects and Underlying Molecular Mechanisms of Long-Term Salt Content Diets in Spontaneously Hypertensive Rats

**DOI:** 10.1371/journal.pone.0141288

**Published:** 2015-10-23

**Authors:** Rebeca Caldeira Machado Berger, Paula Frizera Vassallo, Renato de Oliveira Crajoinas, Marilene Luzia Oliveira, Flávia Letícia Martins, Breno Valentim Nogueira, Daisy Motta-Santos, Isabella Binotti Araújo, Ludimila Forechi, Adriana Castello Costa Girardi, Robson Augusto Souza Santos, José Geraldo Mill

**Affiliations:** 1 Department of Physiological Science—Federal University of Espirito Santo, Vitória, ES, Brazil; 2 Heart Institute (InCor) University of São Paulo Medical School, São Paulo, SP, Brazil; 3 Department of Physiology and Biophysics -Federal University of Minas Gerais, Belo Horizonte, MG, Brazil; 4 Department of Morphology—Federal University of Espirito Santo, Vitória, ES, Brazil; University of Louisville, UNITED STATES

## Abstract

Several evidences have shown that salt excess is an important determinant of cardiovascular and renal derangement in hypertension. The present study aimed to investigate the renal effects of chronic high or low salt intake in the context of hypertension and to elucidate the molecular mechanisms underlying such effects. To this end, newly weaned male SHR were fed with diets only differing in NaCl content: normal salt (NS: 0.3%), low salt (LS: 0.03%), and high salt diet (HS: 3%) until 7 months of age. Analysis of renal function, morphology, and evaluation of the expression of the main molecular components involved in the renal handling of albumin, including podocyte slit-diaphragm proteins and proximal tubule endocytic receptors were performed. The relationship between diets and the balance of the renal angiotensin-converting enzyme (ACE) and ACE2 enzymes was also examined. HS produced glomerular hypertrophy and decreased ACE2 and nephrin expressions, loss of morphological integrity of the podocyte processes, and increased proteinuria, characterized by loss of albumin and high molecular weight proteins. Conversely, severe hypertension was attenuated and renal dysfunction was prevented by LS since proteinuria was much lower than in the NS SHRs. This was associated with a decrease in kidney ACE/ACE2 protein and activity ratio and increased cubilin renal expression. Taken together, these results suggest that LS attenuates hypertension progression in SHRs and preserves renal function. The mechanisms partially explaining these findings include modulation of the intrarenal ACE/ACE2 balance and the increased cubilin expression. Importantly, HS worsens hypertensive kidney injury and decreases the expression nephrin, a key component of the slit diaphragm.

## Introduction

The association of the excessive salt intake with hypertension, cardiovascular and renal diseases is well accepted. Besides its hemodynamic effect, salt overload is believed to promote additional non-pressure-related adverse effects, including cardiac hypertrophy, impaired ventricular relaxation, endothelial dysfunction, increased oxidative stress and renal injury. Together, these effects accelerate glomerular damage, interstitial fibrosis and proteinuria [1–4]. In contrast, dietary salt restriction has beneficial effects on target-organs in hypertension, including kidneys [5–8]. However, the molecular mechanisms underlying such effects have not been fully elucidated. In fact, evidences suggest a direct pathogenic role for high salt intake in renal failure [9], and salt reduction has been shown to decrease proteinuria in kidney disease [10,11]. Given the high salt intake found in most of modern populations, the mechanisms by which high levels of salt intake may contribute to cardiovascular and renal injury, and how low salt acts to avoid these effects are of paramount importance. Although, the benefits of low salt diets in cardiovascular disease events have been recently questioned [12–13].

Both hemodynamic maladjustments and altered proximal tubular function seem to be responsible for triggering renal disease in hypertension. In this regard, a recent study showed that microalbuminuria progression in spontaneously hypertensive rats (SHRs) is associated with reduced expression of key components of the apical endocytic apparatus in the renal proximal tubule, including megalin, cubilin and the H^+^/Cl^-^ exchange transporter 5, ClC-5 [14]. In addition, Bonnet et al. [15] have shown that the expression of the podocyte slit-diaphragm protein nephrin is decreased in an experimental model of hypertension associated with diabetic nephropathy and that the renin-angiotensin system (RAS) could be involved in nephrin down regulation. Although studies have shown that, in some cardiovascular disease, there are changes in the expression of slit-diaphragm proteins and critical components of the endocytic machinery in the renal proximal tubule, the salt influence in the expression of these proteins in hypertension has not been investigated.

It is known that expression and functioning of SRA components are closely related to salt intake. Studies have shown that the blockade of the AT1 angiotensin II receptor prevents cardiovascular and renal effects of a high salt load independent of the blood pressure in SHR [16]. Moreover, salt reduction is recommended in the treatment of hypertension because it produces not only a blood pressure lowering effect *per se* but also contributes to the antihypertensive effects of drugs and enhances the renal protective effect of angiotensin-converting enzyme (ACE) inhibitors [17,18]. However, the molecular mechanisms by which changes in the salt intake interferes with renal function in hypertension is still unclear. Therefore our purpose in this study was to investigate the long-term effects of different salt content diets on the renal function of SHR and to explore potential molecular mechanisms involved in renal damage or protection produced, respectively, by high and low salt diets.

## Materials and Methods

### Animals and groups

Animals were provided by the Central Animal House of the Federal University of Espirito Santo. All protocols of this study were in accordance with the Guidelines for the Care and Use of Laboratory Animals [19] and the Ethical Principles of the Brazilian College of Animal Experimentation (COBEA). The protocols were also previously approved by the Institutional Committee of Ethics on Animal Research and the Institutional Animal Research Committee (Process n°053/2012). Male SHR from the Institutional Animal Facility were divided into three groups fed with a diet that differed only in its sodium content. Experimental diets were introduced just after weaning (four weeks) and maintained for the next 6 months. The three diets tested were isocaloric and isoproteic. The standard normal salt diet (NS) contained 0.3% NaCl. The low salt diet (LS) was prepared by reducing the salt content of the NS diet by 10-fold (0.03% NaCl) to mimic a highly restrictive salt intake, as recommended to patients with chronic kidney diseases. The high salt (HS) diet was prepared with 3% NaCl. All diets were obtained from PragSoluções (São Paulo-SP, Brazil). During this study the animals were maintained in a 12-h light–dark cycle in a controlled-temperature room (22–25°C). Access to water and chow was free along all the experimental period.

### Measurement of systolic blood pressure

Systolic blood pressure (SBP) was measured by tail pletismography at weaning, 3^rd^ and 6^th^ month. Animals were allowed to adapt to the container for 10 to 15 min, and tail-cuff signals from the transducer were automatically collected using an IITC apparatus (IITC Inc., California, USA) connected to a dedicated computer. The measurements and data analysis were performed as previously described [20].

### Renal function parameters

In the last week of treatment protocol, the rats were placed in individual metabolic cages (Tecniplast 304) for 48 hours for the analysis of metabolic parameters. The first 24 hours were used for adaptation, and the following 24 hours were used to record food and water intake and to collect all the urine produced. NS, LS or HS chows were given *ad libitum* to the respective experimental groups. Urine produced in 24 h was measured by gravimetry and samples were immediately frozen at -20°C for posterior analysis. Creatinine concentration in blood and urine was determined by a kinetic method (Labtest, Minas Gerais—Brazil) based on the Jaffé reaction. The glomerular filtration rate (GFR) was estimated by the creatinine clearance. Sodium and potassium excretion was measured on a radiometer ABL800 Flex121 blood gas analyzer (Radiometer Medical, Brønshøj, Denmark). Protein excretion was determined using a spectrophotometric assay with a Sensiprot kit (Labtest, Minas Gerais—Brazil). The concentration of albumin in the urine samples was determined by using an ELISA kit (Nephrat kit Exocell, Philadelphia, PA). All kits were used following the manufacturer’s instructions.

### Urine protein electrophoresis

Urine samples containing 5 μg of creatinine were solubilized in SDS buffer (2% SDS, 10% glycerol, 0.1% bromophenol blue, 50 mM Tris, pH 6.8), and the proteins were separated by SDS-PAGE using 12% polyacrylamide gels. Following electrophoresis, gels containing urine samples were silver stained using the ProteoSilver Plus kit (Sigma-Aldrich Chemical, St. Louis, Missouri, USA) to detect the urinary proteins.

### Collection of blood and kidney samples

At the end of the treatment protocol, the rats were deeply anesthetized with ketamine (70 mg.kg^-1^, *i*.*p*., Agener—União Brazil) and xylazine (10 mg.kg^-1^, *ip*, Bayer, Brazil). The rats were cannulated to collect blood and euthanized by opening the thorax and removing the heart. The blood sample was centrifuged at 4°C for 15 min at 1400 g, and the serum was separated in tubes containing a coagulation activator. The kidneys were removed, decapsulated, quickly weighted and rinsed in cold saline. The serum and the right kidneys were flash-frozen in liquid nitrogen and stored at -80°C for analysis. The left kidneys were processed for histology.

### Histological analysis

The kidneys were fixed in phosphate-buffered 4% paraformaldehyde and embedded in paraffin. Sections of 4 μm were cut, deparaffinized with xylene and then rehydrated with a descending ethanol gradient. Histological sections were stained with Picrosirius red to evaluate the extent of the interstitial fibrosis. The morphological parameters and the glomerulosclerosis score were evaluated in periodic-acid Schiff (PAS) stained slices as previously described [21]. Renal sections were examined under light microscopy (*Olympus Corporation*, *Japan*). The images were acquired in an AxioCam (*Carl Zeiss MicroImaging*, *Germany*) attached camera with 400x magnification and the images were collected with the AxioVision Imaging System 4.8 software (Carl Zeiss MicroImaging, Germany). A total of 20 glomeruli with detectable vascular and urine pole were examined for each rat. The average percentage of the PAS-positive area, which corresponding to the affected regions, was calculated for each rat using the Image J software analysis tool (public domain). Additionally, the areas of the glomeruli and Bowman's capsule were measured. To assess the extent of interstitial fibrosis, 20 images were randomly captured from the cortex, including the proximal tubules. The hot pink color fraction and its intensities in the renal cortex, which is representative of interstitial fibrosis, were quantified by using the Image J software. A single examiner, blinded to the treatment assignment, performed all histological measures.

### Transmission electron microscopy (TEM)

For ultrastructural analysis, the kidney samples from the different groups were fixed in Karnofsky fixative (2.5% glutaraldehyde and 2% paraformaldehyde) and processed for TEM. After washing in 0.1 M sodium cacodylate buffer and post-fixation in 1% osmium tetroxide for 1 h, the samples were dehydrated in ascending grades of acetone (30%, 50%, 70%, 90%, and 100%) and embedded in epoxy resin/EPON (EMbed 812, Electron Microscopy Sciences). Regions of interest were identified in 0.5 μm semi-thin sections stained with toluidine blue. The sections (60–80 nm) were collected on copper grids and doubly contrasted with solutions of uranyl acetate and lead citrate before acquisition of TEM images (JEOL JEM-1400, Japan) operated at 60–70 kV. A single examiner, blinded to the treatment assignment, performed TEM analysis.

### ACE activity

ACE activity in kidney was assessed using a fluorimetric assay as previously described [22]. Briefly, the tissue samples were homogenized in borate buffer (0.4 M, pH 7.2) containing 0.34 M sucrose and 0.9 M NaCl. Homogenates were centrifuged, and the supernatants were used for analysis. Supernatants from the homogenized tissues were incubated with an assay buffer containing 5 mM Hip-His-Leu (Sigma-Aldrich) for 30 min at 37°C. The reaction was stopped by the addition of 0.34 N NaOH. Then *o*-phthaldialdehyde (20 mg/mL in methanol) was added to the reaction medium, which binds to the reaction product His-Leu and allows for a fluorimetric read. After 10 min, 3 N HCl was added to acidify the solution and then centrifuged. The supernatants were read in an ELISA Reader (BioTek Synergy TM 2; Biotek, VT, USA) using the following wavelengths (365 nm excitation and 495 nm emission). All assays were performed in duplicate. The protein concentration was determined in all samples by using a standard Bradford assay [23]. ACE activity was expressed as nMol His-Leu/min/μg of protein.

### ACE2 activity

Renal ACE2 enzymatic activity was determined following incubation with a fluorogenic peptide substrate [ACE2 substrate:fluorogenic peptide VI (FPS VI), Mca-YVADAPK(Dnp)-OH (catalogue no. ES007; R&D Systems, Minneapolis, MN, USA)] as previously described [24]. In brief, tissue samples were homogenized in ACE buffer (75 mmol l^−1^ Tris–HCl (pH 7.5), 1 mol l^−1^ NaCl and 0.5 mmol l^−1^ ZnCl_2_). Homogenates were centrifuged, and the resulting supernatants were used for analysis. All assays were performed in duplicate. A reaction mixture containing 0.1 M NaCl and 10 mM captopril in ACE2 buffer was added to each supernatant. Fluorescence was emitted due to the breakdown of the FPS VI peptide and was measured using a microplate reader (BioTekSynergy™ 2; BioTek, Winooski, VT, USA) every minute for 120 min immediately after the addition of the fluorogenic peptide substrate at 37°C (320 nM excitation and 405 nM emission). The total fluorescence was corrected according to the protein content, as determined by the Bradford assay [23]. Data were presented in fluorescence units per minute normalized to the total protein concentration.

### Western blot analysis

Kidney tissues were homogenized in a Potter-Elvehjem-style tissue grinder for 25 strokes in ice cold PBS (10 mM phosphate, 140 mM NaCl, pH 7.2) containing protease (1 μM pepstatin, 1 μM leupeptin, and 230 μM PMSF) and phosphatase inhibitors (15 mM NaF and 50 mM sodium pyrophosphate). Protein concentration was determined by the Lowry method [25] Equivalent protein amounts of kidney proteins were solubilized in SDS sample buffer (2% SDS, 10% glycerol, 0.1% bromophenol blue, 50 mM Tris, pH 6.8). Sample proteins were separated by SDS-PAGE using 7.5 or 10% polyacrylamide gels and transferred to polyvinylidene difluoride membranes (PVDF, Immobilon-P; Millipore) at 500 mA for 5 h at 4°C with a TE 62 Transfer Cooled Unit (GE HealthCare, Piscataway, NJ, USA), stained with Ponceau S in 0.5% trichloroacetic acid, and subjected to immunoblotting. The membrane was blocked for 1 h with 5% non-fat dry milk in TBS containing 0.1% Tween-20 (TBS-T) at room temperature. Each membrane was incubated overnight (4°C) with one of the following primary antibodies: megalin (1:50000, a gift from Dr. Daniel Biemesderfer, Yale University, New Haven, CT USA), cubilin (1:1000), nephrin (1:1000) and podocin (1:1000) (Santa Cruz Biotechnology Inc., Santa Cruz, CA, USA), anti-ACE (1:1000 Santa Cruz, CA, USA), anti-ACE2 (1:1000 Abcam, Cambrige, UK). The membranes were then washed three times for 10 min with TBS-T and incubated for 1 h with horseradish peroxidase-conjugated immunoglobulin secondary antibody (1:2,000). Bound antibody was detected using an enhanced chemiluminescence system (GE Healthcare) according to the manufacturer’s protocols. The membranes were stripped with Restore™ Western Blot Stripping Buffer (ThermoFischer Scientific) and reprobed with actin (1:50,000 Merck, Darmstadt, Germany) for the endogenous control of protein quantification. The signals were captured using an ImageQuant LAS 4000 mini system (GE HealthCare). The results were quantified using Image J software.

### Statistical analysis

Data are shown as mean ± standard error of the mean (SEM). Comparisons among means were assessed using one-way analysis of variance (ANOVA) followed by the Bonferroni post-hoc test for parametric distribution variables. For variables with non-normal distribution, the Kruskal-Wallis test, followed by the Dunn post-hoc test, was used. All statistical analyses were performed with the PRISMA 13.0 package (Chicago, IL, USA). Statistical significance was taken as P < 0.05.

## Results

### Body weight, systolic blood pressure, and kidney morphology

Table 1 shows the values of the body weight and systolic blood pressure during the treatment period as well as some morphological characteristics of the kidneys at the end of treatment. Body weight gain was significantly lower (P<0.05) in the LS group (240 ± 8 g, n = 11) compared to the groups under normal or high salt diets (310 ± 6 g, n = 13 and 326 ± 8 g, n = 11, respectively). However, the left kidney weight/body weight ratio, used as an index of renal hypertrophy, was similar among the three groups after 6 months on diets with different sodium content. SBP significantly increased in the three groups during the 6-month follow-up period. After 6 months of treatment, the high salt diet was associated with a significant increase of the SBP when compared to the other two groups (NS: 208 ± 4; LS: 166 ± 3; HS: 227 ± 7 mmHg p < 0.05). However, the low salt diet significantly attenuated the blood pressure increase, without complete normalization, over the entire period when compared to the other groups.

**Table 1:** Morphologic characteristics of the kidneys.

	NS (n = 13)	LS (n = 14)	HS (n = 11)
Body Weight (g)	310 ± 6	240 ± 8* +	326 ± 8
Kidney (mg)	1250 ± 84	908 ± 33* +	1307 ± 38
Kidney/BW (mg/g)	4.0 ± 0.2	3.8 ± 0.1	4.0 ± 0.1
SBP at weaning (mmHg)	130 ± 5	-	-
SBP 3^rd^ month (mmHg)	184 ± 2	153 ± 5 * +	182 ± 6
SBP 6^th^ month (mmHg)	208 ± 4	166 ± 3 * +	227 ± 7* #
Glomerular area (μm^2^)	7182 ± 335	6223 ±141	8791 ± 336* #
Bowman's Capsule area (μm^2^)	8927 ± 260	7887 ± 221	10960 ± 429* #
Bowman's Space area (μm^2^)	1771 ± 145	1664 ± 129	2188 ± 130#

Values are mean ± SEM. SBP: systolic blood pressure. NS: normal salt diet. LS: low salt diet. HS: high salt diet. SBP at weaning: subsample of 8 rats.

* P<0.05 vs. NS

+ P < 0.05 vs. HS

# P < 0.05 vs LS.

(n = 6 / group for histological parameters).

### Urinary protein and albumin excretion

**Fig 1:**
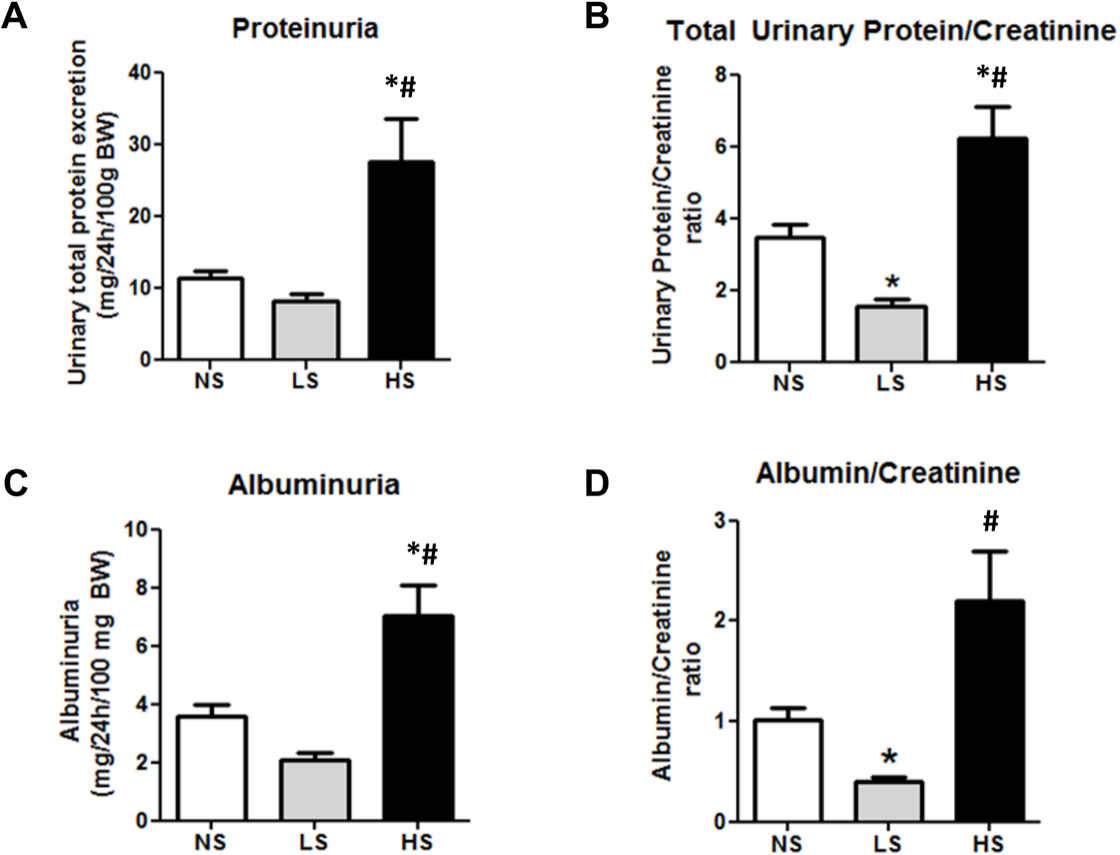
Analysis of the renal function in SHR. (A, B) proteinuria and urine protein/creatinine ratio. (C, D) Albuminuria and albumin/creatinine ratio. The analyses were performed with 24-h urine samples. Data are means ± SEM. NS: normal salt diet. LS: low salt diet. HS: high salt diet. *P<0.05 vs. NS; # P < 0.05 vs LS. (NS n = 10, LS n = 14, HS n = 11).

**Fig 2:**
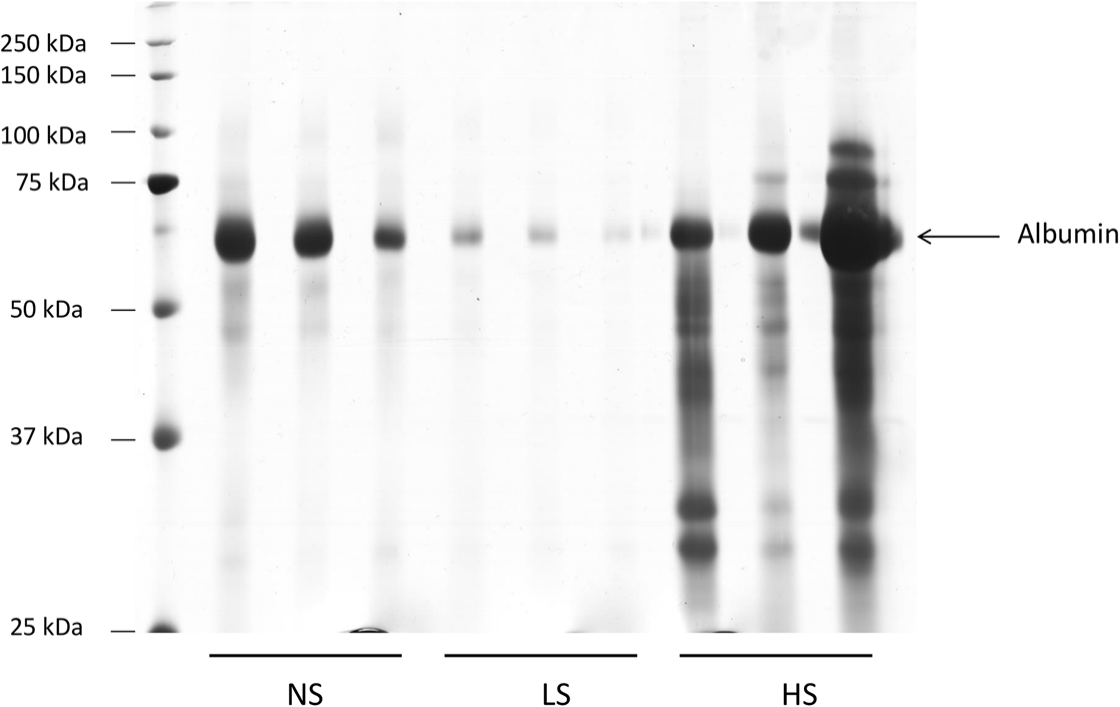
Evaluation of the urinary protein loss. 24-h urine samples from SHRs were subjected to 12% SDS-PAGE and silver stained. NS: normal salt diet. LS: low salt diet. HS: high salt diet.

As expected, the 24-h water intake, urinary output and urinary sodium excretion were strongly affected by the salt content on diets, without any influence on the GFR and sodium concentration in serum (Table 2). The urinary loss of potassium was also similar among the three groups. However, after 6 months on experimental diets, the urine analysis by ELISA and spectrophotometry revealed a pronounced increase in albuminuria (NS: 3.6 ± 0.4; LS: 2.1 ± 0.3 HS: 7.0 ± 1.1 mg/24h/100g BW, p < 0.001) and proteinuria (NS: 11 ± 1; LS: 8 ± 1 HS: 28 ± 6 mg/24h/100g BW, p < 0.001) in the animals under the high salt diet. On the other hand, protein and albumin loss was attenuated in the group under the low salt diet, mainly when protein loss was adjusted to the creatinine excretion (Fig 1). The urine electrophoreses also revealed the presence of high molecular weight proteins in the animals fed with the high salt diet, suggesting the presence of glomerular injury in these animals and a decreased of proteinuria in LS diet (Fig 2).

**Table 2:** Parameters of renal function.

	NS (n = 12)	LS (n = 14)	HS (n = 14)
Chow intake (g/24 h)	17 ± 1	17 ± 1	16 ± 1
Water intake (mL/24 h)	35 ± 1	27 ± 1	77 ± 4* #
Urinary output (mL/24 h/100 g)	6.2 ± 0.4	5.0 ± 0.3	26.1 ± 1.5* #
Serum creatinine (mg/dL)	0.50 ± 0.02	0.47 ± 0.02	0.47 ± 0.01
Urinary Creatinine (mg/mL)	3.9 ± 0.56	5.2 ± 0.38	4.8 ± 0.52
GFR (mL/min/100 g)	0.60 ± 0.1	0.78 ± 0.1	0.71 ± 0.1
Urinary Na^+^ (mEq/24 h/100 g)	0.20 ± 0.01	0.03 ±0.01* ^+^	7.1 ± 0.5* #
Urinary K^+^ (mEq/24 h/100 g)	1.2 ± 0.1	1.2 ± 0.1	1.7 ± 0.3
Serum Na^+^ (mEq/L)	138 ± 1	136 ± 2	141 ± 1

Values are represented as the mean± SEM. NS: normal salt diet. LS: low salt diet. HS: high salt diet. GFR: glomerular filtration rate

*P<0.05 vs. NS

+ P < 0.05 vs. HS

# P < 0.05 vs LS.

### Expression of glomerular filtration barrier components and apical endocytic apparatus receptors

**Fig 3:**
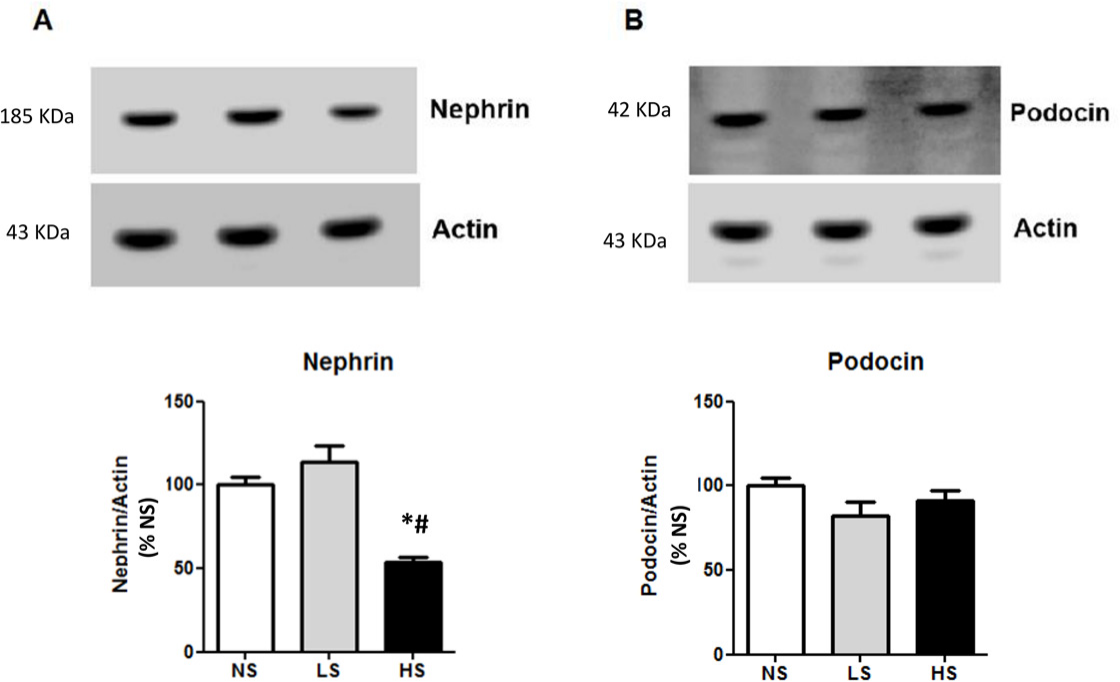
Analysis of nephrin and podocin renal protein expression. (A) Nephrin. (B) Podocin. Data are means ± SEM. NS: normal salt diet. LS: low salt diet. HS: high salt diet. *P<0.05 vs. NS; # P < 0.05 vs LS. (N = 6 / group).

**Fig 4:**
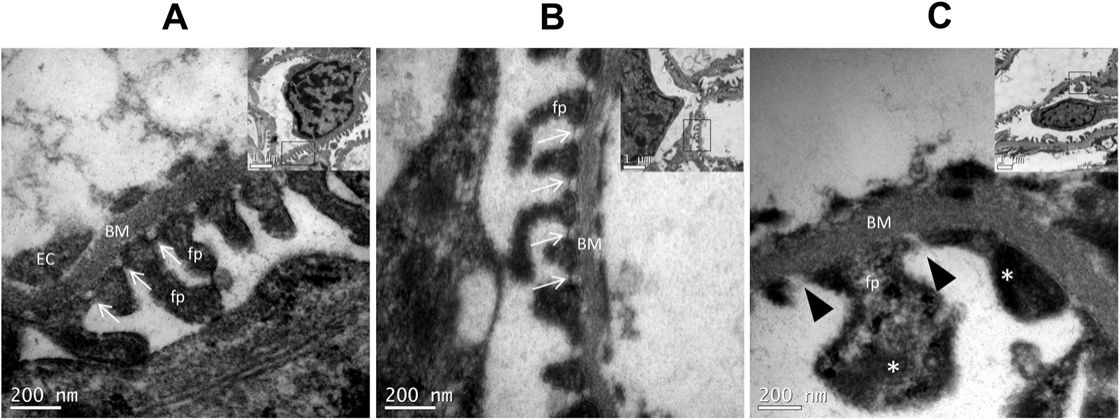
Glomeruli ultrastructural examination. Transmission electron microscopy images show the ultrastructural pedicels (fp) and slit diaphragms (SD, arrows) from the podocytes. Images A and B show the normal podocyte structure from Normal and Low salt group respectively, with SD integrity. Image C shows that the high salt group has lost the morphological integrity of its podocyte processes (fusion/effacement*) along with the slit diaphragms (black arrow head). BM, basement membrane; EC, endothelial cell.

**Fig 5:**
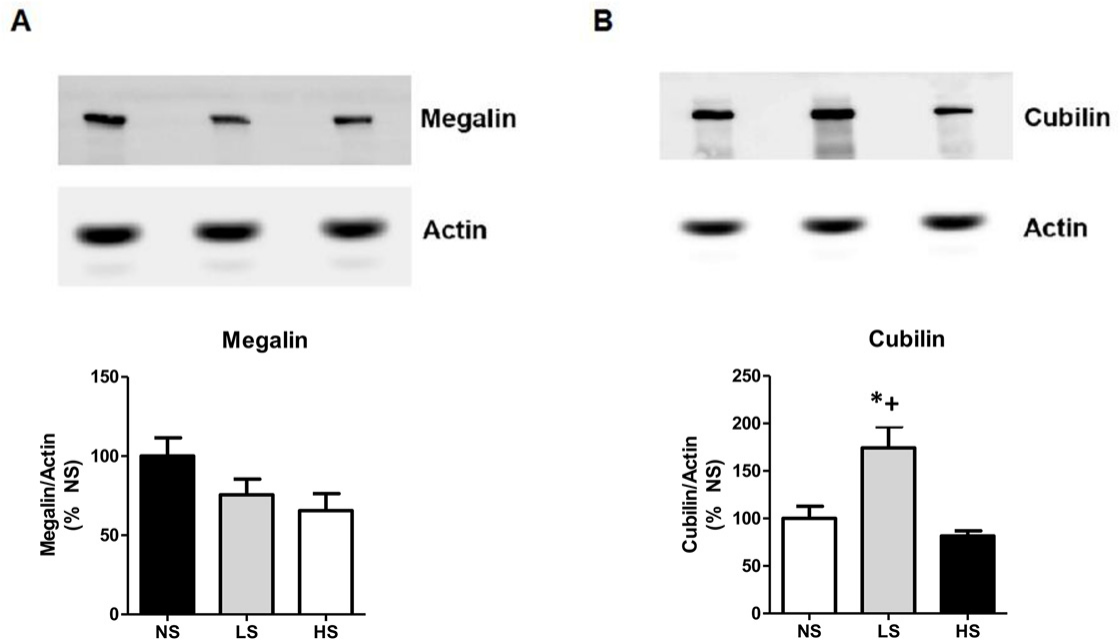
Analysis of megalin and cubilin renal protein expression. (A) Megalin. (B) Cubilin. Data are means ± SEM. (NS) normal salt diet. LS: low salt diet. HS: high salt diet. *P<0.05 vs. NS; + P < 0.05 vs. HS. (n = 6 / group).

Because our results suggested the presence of glomerular proteinuria in SHR fed with the HS diet, we decided to investigate the expression of nephrin and podocin, two proteins of the slit diaphragm domain of podocytes, which represent a selective filtration barrier for the majority of proteins. Fig 3A shows that the expression of nephrin was significantly reduced in the HS group compared to the LS and the NS groups. Additionally, the transmission electron microscopy images showed that these animals also had an observable loss in the morphological integrity of the podocyte processes along with a loss of the slit diaphragm (Fig 4). Additionally, we evaluated the protein expression of the endocytic receptor megalin and its intracellular partner cubilin, both of which are important for the proximal tubular clearance of most proteins filtered in the glomeruli, especially albumin. As detected by western blot, the LS diet increased the protein expression of the endocytic receptor cubilin. This adaption may contribute to reduce the urinary loss of protein in the animals receiving low quantities of sodium in diet (Fig 5B).

### Glomerular and interstitial changes

**Fig 6:**
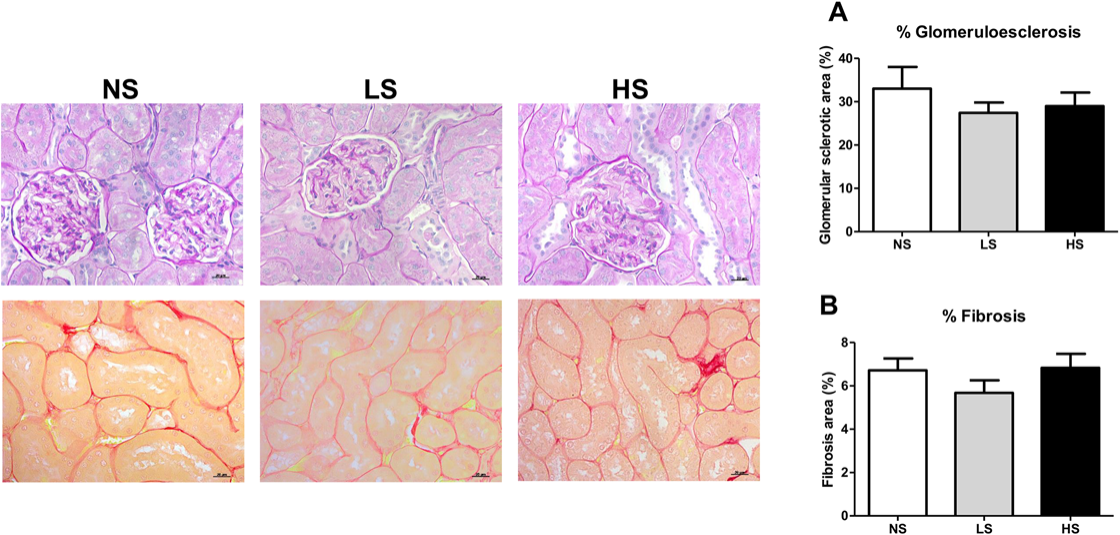
Histological analysis. (A) Photomicrographs of kidney sections stained with Periodic Acid Schiff (PAS) to determine glomerulosclerosis score (percentage of the PAS-positive in glomerular tuft area) and morphometric changes in the groups. (B) Photomicrographs of kidney sections stained with Picrosirius Red to determine interstitial collagen deposition. Data are represented as mean ± SEM. NS: normal salt diet. LS: low salt diet. HS: high salt diet. Scale bar: 20 μm.

Morphometric kidney parameters and glomerulosclerosis score were investigated in the PAS-stained slices. The histological analysis showed clear signs of glomerular hypertrophy in the HS group, as assessed by measuring the glomerular and Bowman's capsule areas and the dilation Bowman's space (Table 1). Additionally, extensive protein cast formation was observed in the animals subjected to long-term high salt diet. These abnormalities may be associated with damage of the ultrafiltration barrier and the down regulation of nephrin, leading to the increased urinary excretion of high molecular weight proteins. The glomerulosclerosis score and extent of the interstitial fibrosis stained with Picrosirius red, however, were unaffected by changes in the salt content of the diets (Fig 6A and 6B, respectively).

### Intrarenal renin angiotensin system components

**Fig 7:**
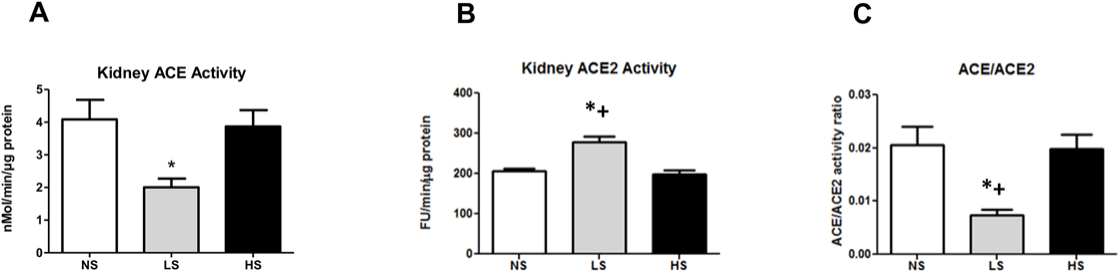
Renal enzymatic activity of ACE, ACE2 and the ACE/ACE2 ratio. (A) ACE enzymatic activity. (B) ACE2 enzymatic activity. (C) ACE/ACE2 ratio. Data are means ± SEM. NS: normal salt diet. LS: low salt diet. HS: high salt diet. *P<0.05 vs. NS; + P < 0.05 vs. HS. (NS n = 12, LS n = 10, HS n = 9).

**Fig 8:**
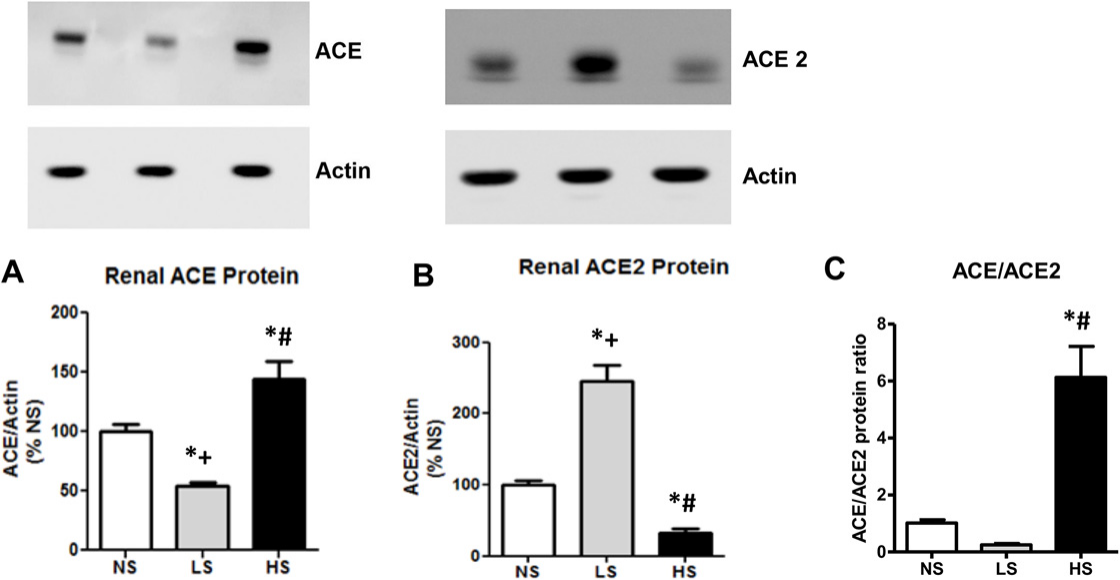
Analysis of ACE, ACE2 expression and ACE/ACE2 protein ratio. (A) ACE kidney expression. (B) ACE2 kidney expression. C: ACE/ACE2 protein ratio. Data are means ± SEM. NS: normal salt diet. LS: low salt diet. HS: high salt diet. *P<0.05 vs. NS; + P < 0.05 vs. HS; # P < 0.05 vs LS.

The RAS is a pivotal mediator of renal homeostasis. ACE is the main component responsible for Ang II synthesis while ACE2 is mainly responsible for Ang-(1–7) generation. ACE2 has recently emerged as a possible RAS component responsible for renal protection in several diseases [21,24]. As illustrated in Fig 7, SHR under LS diet vs. NS diet showed an increased ACE2 activity and a decreased ACE activity in the kidney which resulted in a significant reduction of the ACE/ACE2 ratio. The kidney expression of both enzymes was consistent with this finding (Fig 8). Surprisingly, as compared to NS, the HS diet significantly increased ACE and decreased ACE2 expression which resulted in an increase in the ACE/ACE2 protein ratio, with no change in the activity of both enzymes.

## Discussion

The kidney is an important target organ in hypertension pathophysiology. Moreover, it is well known that high salt intake strongly contributes to hypertension development and to its complications, including chronic kidney disease [26]. Excessive salt intake alone has been associated with renal injury. Thus, salt restriction is recommended for patients suffering from either hypertension or kidney diseases [17, 27]. However, the association between sodium intake, blood pressure and renal injury, and their related mechanisms, are poorly understood. This study showed that a long-term low salt diet (6 months) was able to attenuate the spontaneous hypertension in SHR with a clear renoprotective effect, leading to a reduction in proteinuria and albuminuria. Moreover, we showed for the first time that this effect was associated with an increase in cubilin expression, in parallel with increased ACE2 activity and a reduction in the ACE activity in the kidney. On the other hand, we found that the high salt diet worsened the hypertension, accelerated the renal damage and increased kidney ACE/ACE2 protein ratio, as previously reported [28]. Indeed, excessive long-term salt intake in SHRs causes albuminuria and apparently glomerular proteinuria with reduced expression of nephrin, which was accompanied by glomeruli hypertrophy.

Other studies were previously performed on this subject, however, almost all, tested diets with much higher sodium content (generally 8% of Na in chow or 1% NaCl in drinking water) and for a shorter period of time [1, 29, 30]. These very high salt diets promote not only functional damage and renal fibrosis in hypertensive models but also in normotensive rats as well. We did not detect this change in our study in SHRs, possibly due to lower levels of salt in the HS diet, which may have allowed the kidneys to adapt to the chronic salt overload. Unlike other studies, in which the kidney damage was assessed after short-term exposure with a very high salt intake, we exposed hypertensive rats to a long-term with a moderate increase in the dietary salt content.

Previous studies have shown that development of microalbuminuria in SHRs seems to be predominantly tubular, with the urinary loss of low molecular weight proteins [14, 31]. Here, we observed that long-term high salt intake was associated with the urinary loss of proteins with a high molecular weight, suggesting that damage to the ultrafiltration barrier in turn leads to glomerular proteinuria. Importantly, it was confirmed by ultrastructural evidence that the high salt group lost the morphological integrity of the podocyte processes and the slit diaphragms. The observed decrease in the nephrin component of the podocytes in the HS diet group may help to explain these findings. The glomerular filtration barrier consists of a fenestrated endothelium, a basement membrane and the outermost podocyte foot process with their slit diaphragms, which is the ultimate barrier for proteins. It is now known that this barrier is primarily composed of the nephrin protein [32]. Mutations in the gene encoding nephrin are known to result in proteinuria and nephrotic syndrome [33]. A down-regulation of the expression of nephrin has been demonstrated in SHR when compared to normotensive controls [34]. We found an additional decrease in nephrin expression in SHR subjected to a HS diet, which may contribute to accelerate the glomerular damage resulting in the hyperfiltration of plasma proteins. On the other hand, we could see in the analysis of urine samples that the long-term low salt intake was able to decrease the proteinuria in the SHR to a level even lower than that observed in control rats receiving a normal salt diet, which were associated with the increase in cubilin expression.

To our knowledge, this is the first demonstration that dietary salt reduction alone can prevent proteinuria in SHRs associated with a higher renal cubilin expression. This finding indicates the unequivocal beneficial effect of the long-term reduction in salt intake in hypertensive disease. Cubilin is a multiligand endocytic receptor in the renal proximal tubule. With its partner the megalin receptor, it forms a complex that is largely responsible for the proximal tubular reabsorption of most proteins filtered in the glomeruli. A recent study showed that the protein expression of megalin and cubilin is decreased in SHRs from 14 to 21 weeks of age [14]. Here, we followed SHRs from weaning until 28 weeks of age and we observed that these animals when receiving the low salt diet displayed an improvement in their cubilin expression as compared to other SHRs groups. We thus suggest that the proteinuria reduction promoted by the low salt intake could be involved with an increase in the capacity for protein reabsorption in the proximal tubule in hypertensive rats.

Unfortunately, with the present data, we cannot clearly separate the beneficial effects of dietary salt restriction on renal system in SHR from the effects produced by the attenuation of hypertension development in these animals. However, several reports favor the hypothesis of a pressure-independent influence of dietary salt restriction on the cardiovascular system [5–7, 35], especially for the renal function. In fructose-induced renal alterations, low salt intake prevented the albuminuria, the kidney inflammation and oxidative stress, without affecting blood pressure, thus suggesting that the beneficial renal effects in this case was unrelated to unrelated to systemic hemodynamic alterations [35].

It remains unclear how salt intake modulates the receptors involved in protein filtration and reabsorption in the kidney. It is possible to speculate that the RAS is related to these mechanisms. Indeed, it is well known that agents targeting classical components of the RAS exert renoprotective effects beyond their blood pressure lowering effect [36–38], especially regarding the improvement of renal albumin handling [12,39]. In the human glomerulonephritis for example, the long-term antiproteinuric effect of the ACE inhibitor lisinopril could not be acutely reversed by angiotensin II infusion, despite a dose-related fall in renal plasma flow and increase in systolic blood pressure, filtration fraction and renal vascular resistance [40]. Recently, Arruda-Junior et al. [39] showed that the reduction of tubular proteinuria in SHRs treated with losartan, an AT1 blocker, was accompanied by an increased renal megalin expression and that these beneficial effects were independent of blood pressure reduction. Additionally, studies performed in an experimental model of hypertension and diabetes have shown that treatment with the angiotensin II antagonist irbesartan prevented the development of albuminuria and the down-regulation of nephrin expression, without a prominent influence on blood pressure levels [15]. Collectively, our current findings, together with those on literature, suggest that high salt may activate intrarenal RAS and alter the expression of slit-diaphragm proteins and critical components of the endocytic machinery in the renal proximal tubule, which in turn leads to proteinuria. Conversely, we speculate that low salt diet may mitigate the intrarenal RAS activation in pathological states, including hypertension, and therefore preserve renal handling of albumin.

In the present study, the long-term use of a diet with a high salt content significantly increased the kidney ACE/ACE2 protein ratio, which seems to be one of the mechanisms by which a high salt diet leads to renal damage, most likely by increasing the Ang II/ Ang-(1–7) ratio. A similar finding has been recently reported [28]. Curiously, in the high salt group, ACE/ACE2 activity ratio did not follow the increase of ACE/ACE2 protein ratio. There are some possible explanations for these findings. First, the techniques currently available to measure ACE and ACE2 activities generally need supraphysiological concentrations of synthetic substrates [22,24], which may not reflect the real endogenous enzyme activity. Second, previous studies reported the presence of endogenous inhibitors of circulating ACE [41] and ACE2 [42] upon which ACE and ACE2 activities are suppressed *in vivo*. Indeed, Varagic et. al [43] observed similar findings to ours regarding ACE2 protein and activity. In note, the uncoupling among ACE2 gene, protein, and activity has already been reported [44–46]. Therefore in future studies it would be important to measure the local levels of Ang II and Ang-(1–7) in order to clarify it.

Conversely to that observed in a high salt diet, we found that SHRs subjected to the low salt had a clear change in both protein and enzyme activity. In this condition we found an increase in kidney ACE2 protein and activity when compared to the other SHR groups. We also found a decline in ACE protein and activity, which resulted in a clear decrease in the ACE/ACE2 protein ratio in parallel to a similar change in the enzymatic activities. The long-term low salt diet thus seems to modulate intrarenal SRA and thereby interferes simultaneously with ACE and ACE2 activity in kidney tissues via the ACE2 pathway. These changes could decrease the renal production of Ang II in parallel with a higher production of Ang-(1–7). This pattern of intrarenal ACE/ACE2 balance may contribute to the renoprotection found in our study by reducing the salt intake. ACE2 is a primary enzyme that endogenously forms Ang-(1–7) from Ang II, and it has emerged as having an important role for counter-balancing the vasoconstrictor arm of the RAS, mainly dependent of the action of angiotensin II acting at AT1 receptors. It was already shown that the deletion of the ACE2 gene in mice leads to the development of angiotensin II-dependent glomerulosclerosis [21]. ACE2 expression is decreased in the diabetic kidney, which is associated with tubular injury [47], and in hypertensive rats when compared with normotensive controls [48]. Thus, interventions to augment the ACE2 expression or activity have been shown to be useful to prevent cardiovascular damage [49]. It has already been demonstrated that ACE2 can both increase the Ang-(1–7) levels and reduce the kidney angiotensin II levels [50–51]. Thus, for the same reason, it is conceivable that the induction of ACE2 seems to be also protective. In fact, ACE2 kidney expression has been shown to be present in the podocyte/slit diaphragm complex [52], and increases in its activity could be protective facing the decrease of nephrin expression observed with HS diets beyond preserving the cubilin expression. However, further studies are required to elucidate the precise mechanisms involved in the interaction of sodium intake and the RAS in the hypertensive kidney injury. A better understanding of these mechanisms may be valuable for development of new successful therapeutic interventions.

In summary, our study showed that a long-term low salt intake attenuates hypertension progression in SHRs, prevents proteinuria with increase in cubilin expression and is associated with a modulation of the local kidney RAS toward renal protection. In contrast, the high salt diet worsened hypertension, leading to renal injury as demonstrated by the increase in the urinary excretion of albumin and high molecular weight proteins and glomerular damage, all of which are associated with the lower expression of key components of the slit diaphragm protein nephrin and with the increased ACE/ACE2 protein ratio.

It is tempting to speculate that in the LS diet, the local kidney RAS, specifically the ACE2/Ang1-7 axis, may be the pathway that modulates the components of the endocytic apparatus in the renal proximal tubule. This could explain the observed relationship between a low salt intake and renal protection in hypertension.
